# Pemphigus autoimmunity: Hypotheses and realities

**DOI:** 10.3109/08916934.2011.606444

**Published:** 2011-09-23

**Authors:** Sergei A Grando

**Affiliations:** Department of Dermatology, University of California, Irvine, CA, USA

**Keywords:** Pemphigus vulgaris, Pemphigus foliaceus, autoantigen, autoantibody, apoptolysis, prednisone

## Abstract

The goal of contemporary research in pemphigus vulgaris and pemphigus foliaceus is to achieve and maintain clinical remission without corticosteroids. Recent advances of knowledge on pemphigus autoimmunity scrutinize old dogmas, resolve controversies, and open novel perspectives for treatment. Elucidation of intimate mechanisms of keratinocyte detachment and death in pemphigus has challenged the monopathogenic explanation of disease immunopathology. Over 50 organ-specific and non-organ-specific antigens can be targeted by pemphigus autoimmunity, including desmosomal cadherins and other adhesion molecules, PERP cholinergic and other cell membrane (CM) receptors, and mitochondrial proteins. The initial insult is sustained by the autoantibodies to the cell membrane receptor antigens triggering the intracellular signaling by Src, epidermal growth factor receptor kinase, protein kinases A and C, phospholipase C, mTOR, p38 MAPK, JNK, other tyrosine kinases, and calmodulin that cause basal cell shrinkage and ripping desmosomes off the CM. Autoantibodies synergize with effectors of apoptotic and oncotic pathways, serine proteases, and inflammatory cytokines to overcome the natural resistance and activate the cell death program in keratinocytes. The process of keratinocyte shrinkage/detachment and death via apoptosis/oncosis has been termed apoptolysis to emphasize that it is triggered by the same signal effectors and mediated by the same cell death enzymes. The natural course of pemphigus has improved due to a substantial progress in developing of the steroid-sparing therapies combining the immunosuppressive and direct anti-acantholytic effects. Further elucidation of the molecular mechanisms mediating immune dysregulation and apoptolysis in pemphigus should improve our understanding of disease pathogenesis and facilitate development of steroid-free treatment of patients.

## Introduction

Autoimmune pemphigus is a life-threatening mucocutaneous blistering disease associated with IgG antibodies targeting several types of keratinocyte antigens and eliciting epidermal clefting (acantholysis) via intracellular signaling activating apoptotic enzymes (apoptolysis) [1]. Systemic administration of glucocorticosteroid hormones is essential to establish control of disease during the acute stage [2]. Although glucocorticosteroid treatment is life-saving, it may cause severe side effects, including death [3,4]. Therefore, pemphigus patients need drugs that can replace glucocorticosteroids. The development of non-steroidal treatment has been hampered by a lack of clear understanding of the mechanisms leading to keratinocyte detachment and death in pemphigus. This overview of recent advances in the knowledge of pemphigus autoimmunity challenges the existing dogmas, helps resolve old controversies, and identifies new perspectives for treatment. It encompasses knowledge on pemphigus vulgaris (PV) and pemphigus foliaceus (PF), but specifically excludes reports on paraneoplastic pemphigus, or PNP, originally described by Anhalt et al. [5], because this disease is not related to PV and PF. The notion that PNP represents a variant of classical pemphigus stems from the facts that patients with PV or PF sometime have concomitant neoplasms [6-8] and that some patients with PNP develop anti-desmoglein (Dsg) 1 and/or 3 antibodies—the hallmark of classical pemphigus [9]. In fact, PNP represents only one manifestation of the heterogeneous autoimmune syndrome—termed paraneoplastic autoimmune multiorgan syndrome (PAMS)—targeting both tegumental epithelium and internal organs [10]. In marked contrast to classical pemphigus, PAMS has an overall mortality more than 90% despite therapy, with progressive respiratory failure with clinical features of bronchiolitis obliterans being the most frequent cause of death [11]. Sloughing of bronchial epithelial cells contributes to the occlusion of the small airways that provides a potential mechanism for the respiratory failure [10]. Patients with PAMS develop mucocutaneous lesions that resemble pemphigoid, erythema multiforme, lichen planus, and graft vs. host disease, as well as the pemphigus-like variant that was termed PNP in the index patient with PAMS. Oral mucosal lesions of painful, progressive stomatitis are the hallmark of the disease and usually are the initial manifestation of PAMS [12]. The proposed pathogenesis of PAMS continues to evolve. It is clear that the immunopathologic mechanisms differ appreciably from those responsible for the lesions of classical pemphigus. The spectrum of PAMS includes patients with disease predominantly or exclusively mediated by the cellmediated autoimmunity effectors and those with both autoantibodies and cellular cytotoxicity [13].

## Pemphigus autoantigens

Following the discovery of IgG autoantibodies in patients with PV [14] and PF [15], numerous attempts have been made to identify targeted antigens. The patient's serum and isolated IgG fraction were utilized in the immunoprecipitation and immunoblotting experiments using the epidermal or keratinocyte culture proteins as well as saliva and urine as substrates. Although the low-sensitivity approaches, such as fluorography with metabolically labeled keratinocyte proteins preabsorbed with human serum [16], demonstrated single protein bands, a more sensitive but less specific immunoblotting technique revealed more than a dozen of targeted keratinocyte proteins [17]. An enhanced sensitivity immunoprecipitation assay demonstrated that different PV or PF patients produce antibodies recognizing both common and unique antigens [18].

**Figure 1:**
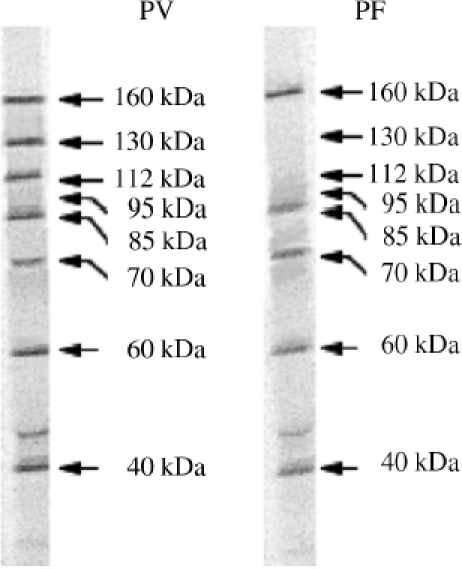
Characterization of anti-keratinocyte antibody profiles of PV and PF sera by immunoprecipitation with proteins from cultures of human epidermal keratinocytes resolved by 7.5% SDS-PAGE. Modified from Ref. [18].

Representative images of protein bands recognized by PV and PF sera are shown in Figure 1. The full list of “pemphigus antigens” reported to date contains over 40 protein bands with apparent molecular weights (MWs) of 12, 18, 25, 30, 33, 35, 38, 40, 45, 47, 50, 52, 55, 57, 59, 60, 62, 66, 67, 68, 70, 75, 78, 80, 85, 95, 100, 102, 105, 110, 112, 120, 130, 140, 160, 170,180, 185/190, 210, and 260 kD [16,18-37]. Indeed, some of these bands may include the same polypeptides migrated with slightly different MWs, and vice versa, some may include two or more similarly sized but distinct antigens. For instance, in addition to the 130 kD Dsg 3, PV patients develop antibodies recognizing yet unidentified protein(s) of the same 130 kD MW in *Dsg3^−/−^* keratinocytes [18] and peripheral blood mononuclear cells [38].

Identification of the nature of proteins targeted by pemphigus autoimmunity is a subject of intense research. Originally, it was assumed that the proteins with the MW of approximately 60 kD or less are “contaminating” keratins that do not represent meaningful targets. However, recent studies demonstrated that only 2% of pemphigus and normal sera contain anti-keratin antibodies [39]. Furthermore, a 66 kD antigen recognized by PV IgG—a membrane glycoprotein composed of two apparently identical subunits of 33 kD—was used to raise rabbit antibody that induced PV-like phenotype in neonatal mouse [27]. Nevertheless, the candidates for the pathophysiologically relevant PV and PF antigens were selected among a few bands migrating with a higher MW, wherein the 130 and 160 polypeptides were most commonly seen [16,29]. The antigens with these MWs were identified as Dsg 3 [17] and Dsg 1 [40], respectively. Thereafter, exploration of the nature of pemphigus antigens has been hampered by a simplistic (or “monopathogenic” [41]) explanation of pemphigus pathophysiology through the “Dsg compensation” hypothesis placing Dsg 1/3 in the center of the pathophysiologic loop [42].

**Figure 2:**
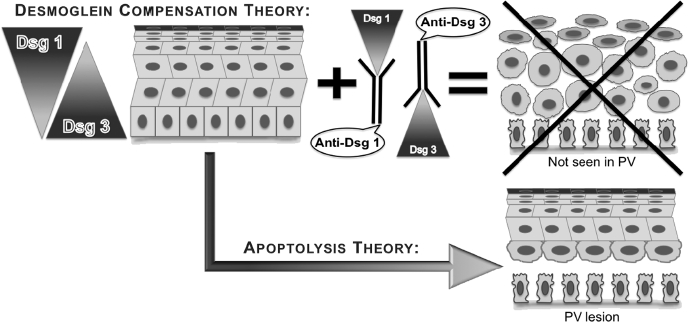
The imaginary appearances of epidermis in the skin of PV patients that produce both Dsg 1 and 3 antibodies based on the postulates of Dsg compensation hypothesis vs. real appearance of lesional epidermis in PV patients.

The Dsg compensation hypothesis maintains that anti-Dsg 1 and 3 antibody profiles in pemphigus sera and the normal epidermal distributions of Dsg 1 and 3 determine the sites of blister formation and that either Dsg 1 or Dsg 3 alone is sufficient to maintain keratinocyte adhesion [42]. The three postulates of this hypothesis are as follows: (1) in the superficial epidermis of PF patients, where Dsg 1 without Dsg 3 is expressed, anti-Dsg 1 antibody alone can cause blisters; (2) Dsg 3 antibody alone is sufficient to cause suprabasal split in the oral mucosa of PV patients that lacks Dsg 1; and (3) skin lesions in PV patients develop when both Dsg 1 and Dsg 3 antibodies are present. The major flaw of this hypothesis is an assumption that the integrity of the stratified squamous epithelium enveloping skin and oral mucosa relies entirely on Dsg 1 and 3 molecules. If that would be the case, the epidermis would have disintegrated to a single cell suspension in the PV patients who develop both anti-Dsg 1 and 3 antibodies (Figure 2).

The monopathogenic explanation of localization of intraepidermal clefts in PV and PF through Dsg compensation hypothesis ignores the complexity of homo- and heterophilic interactions of seven known desmosomal cadherins, i.e. Dsg 1-4 and desmocollin (Dsc) 1-3. In reality, Dsg 3 alone cannot sustain epidermal cohesion. This is evident from the facts that Dsg 3 cannot compensate for a loss of Dsc 3 in the conditional *Dsc3^null^* mutant mouse that exhibits suprabasal acantholysis and overt skin blistering [43]. Furthermore, the *in vitro* experiments demonstrated that extracellular domain of Dsg 3 mediates only a weak homophilic adhesion [44]. Lack of skin blisters in patients with striate palmoplantar keratoderma featuring a deletion mutation in the extracellular domain of Dsg 1 and in mice with engineered or spontaneous mutations of Dsg 3 (reviewed in [45,46]) clearly indicate that the integrity of epidermis does not depend solely on Dsg 1 and 3. The electron microscopic studies demonstrated that keratinocytes deprived of endogenous production of Dsg 1 or 3 due to gene silencing via RNA interference continue to form desmosomes [47]. Apparently, the redundancy of desmosomal cadherins renders the external integument sufficient integrity and durability.

The first evidence that keratinocyte antigens other than Dsg 1 and 3 are pathophysiologically relevant in pemphigus was provided by experiments showing the ability to induce suprabasal acantholysis and gross skin blisters in *Dsg3* neonates by passive transfer of PV patients’ antibodies [48]. In this model, murine epidermis lacked Dsg 3 and the passively transferred PV IgGs lacked anti-Dsg 1 antibody. Hence, the injected PV antibodies that caused blisters could target only the non-Dsg 1 and 3 antigens that mediated and/or regulated keratinocyte adhesion. This observation prompted further investigations into the nature of pemphigus antigens (reviewed in [41]). By now, over 50 human proteins have been reported to specifically react with pemphigus IgG (Table I). In addition to the known desmosomal cadherins and several other types of adhesion molecules, the hitherto identified pemphigus antigens include cell membrane (CM) receptors, immunologic/hematologic antigens, neuronal/oncologic antigens, and thyrogastric cluster antigens.

**Table I:** Self-antigens recognized by pemphigus IgGs.

Antigen	Reference
Adhesion molecules	
Collagen XVII (a.k.a. BPAG2)	[340]
Desmocollin 1	[124]
Desmocollin 2	[124]
Desmocollin 3	[125]
Desmoglein 1	[40]
Desmoglein 2	[341]
Desmoglein 3	[17]
Desmoglein 4	[342]
Desmoplakin 1	[343]
Desmoplakin 2	[343]
E-cadherin	[344]
Intercellular adhesion molecule 1	[49]
Plakoglobin (a.k.a. γ-catenin)	[345]
Plakophilin-3	[346]
Platelet/endothelial cell adhesion molecule	[49]
Cell membrane receptors	
Acetylcholine receptor	[48]
Acetylcholine receptor, Mj muscarinic	[60,61]
Acetylcholine receptor, M_2_ muscarinic	[61]
Acetylcholine receptor, M_4_ muscarinic	[61]
Acetylcholine receptor, M_5_ muscarinic	[61]
Acetylcholine receptor, α3 nicotinic	[57]
Acetylcholine receptor, α9 nicotinic	[36]
Acetylcholine receptor, α10 nicotinic	(Unpublished)
Acetylcholine receptor, ε nicotinic	(Unpublished)
Annexins	[63]
FceRIa	[347]
Neuronal voltage-gated K^+^ channel	[348]
Pemphaxin [a.k.a. annexin 9)	[62]
Taurine transporter-like molecule	[348]
Thrombospondin receptor	[49]
TNF receptor superfamily member 5	[49]
Transmembrane 4 superfamily (a.k.a. tetraspanin family; CD37)	[49]
PERP	[50]
Parathyroid hormone 1 receptor	[50]
TGF-(3 receptor-associated protein	[50]
Insulin-like growth factor 1 receptor	[50]
Immunologic/hematologic antigens	
Hemoglobin ε 1	[49]
Immunoglobulin heavy-chain constant region 72 (Fc-IgG_2_)	[49]
Interferon regulatory factor 8	[49]
Interleukin 1 receptor accessory protein-like 2	[49]
Sialic acid binding Ig-like lectin 3 (CD33)	[49]
Signaling lymphocytic activation molecule 5 (CD84)	[49]
T-cell surface antigen (CD2)	[49]
Neuronal/oncologic antigens	
Carcinoembryonic antigen-related cell adhesion molecule 6	[49]
NADH dehydrogenase-like protein	[348]
Neuronal cytoplasmic collapsin response mediator protein 5	[348]
Nicotinamide/nicotinic acid mononucleotide adenylyltransferase 2	[49]
Unclassified neuronal antigen	[348]
Peripheral myelin protein 22	[49]
Thyrogastric cluster antigens	
Gastric parietal cell antigen	[348]
Glutamic acid decarboxylase (GAD65)	[348]
Proline dehydrogenase 1	[50]
Microsomal antigen	[348]
Thyroperoxidase	[349]

Of particular interest is a recently discovered autoimmunity against a novel member of the peripheral myelin protein (PMP)-22/gas3 family termed PERP as well as the structurally related PMP-22 [49,50]. Knockout mice lacking PERP display a phenocopy of PV [51], which gave rise to a notion that the biologic function of PERP is limited to desmosomal stabilization [52]. However, PERP is expressed in various types of cells that do not form desmosomes [53], which argues against its exclusive biologic function in desmosomal adhesion. Recent findings and its putative tetraspan transmembrane topology implicate a role for PERP in the extrinsic apoptotic pathway that involves direct interaction between adaptor proteins and the receptor complexes activating caspase 8, i.e. PERP is a novel cell death receptor [54]. Hence, dissolution of desmosomes and PV-like intraepidermal split in *Perp* mice apparently result from aberrant inside-out signaling along the altered cell death pathways.

In a radioimmunoprecipitation assay of 34 PV and 6 PF serum, 85% of patients precipitated keratinocyte acetylcholine (ACh) receptors (AChRs) [48]. Noteworthy, human keratinocytes express AChRs of both muscarinic (mAChR) and nicotinic (nAChR) classes, and these receptors regulate cell adhesion in a synergistic fashion (reviewed in [55,56]). Blocking of either class of keratinocyte AChRs leads to disassembly of desmosomal and adherence junctions due to phosphorylation of desmosomal and classical cadherins, respectively, whereas cholinergic agonists prevent cell detachment by activating protein phosphatases and upregulating expression of the cadherin genes. Targeting of a3 nAChR by pemphigus antibody was discovered in a patient with coexistent PF, myasthenia gravis, and thymoma [57]. Indeed, pemphigus patients occasionally develop myasthenia gravis, an anti-nAChR autoimmune disease (reviewed in [6]). Based on the epitope-spreading model [58], patients with myasthenia gravis might produce antibodies binding other members of an overall homologous nAChR protein gene family (reviewed in [59]).

The types of other AChRs targeted by pemphigus autoimmunity have been investigated using various experimental approaches. Reactivity of PV IgG with the mixed muscarinic and nicotinic σ9 AChR was observed in the immunofluorescence blocking experiments, wherein staining of monkey esophagus by rabbit anti-α9 antibody was prevented due to preincubation of the substrate with PV antibodies [36]. Recently, the proteomics approach has demonstrated that PV antibodies react with α10 subunit that can be a part of the pentameric α9α10 nAChR (unpublished). Therefore, the binding site for α9 antibody in the heteromeric α9α10 nAChR also can be hindered by anti-α 10 antibody present in PV sera. When added to keratinocyte monolayer, anti-α9 antibody produced acantholysis [36], indicating that the alteration of either α9 or α10 subunit inactivates functioning of the α9α10 channel coupled to the regulation of keratinocyte adhesion.

Using the proteomics technology, two research groups have independently demonstrated that pemphigus autoimmunity targets the M_1_ subtype of keratinocyte mAChRs [60,61]. The protein array technology also identified M_2_, M_4_, and M_5_ mAChR subtypes as targets of pemphigus autoimmunity [61]. Probing of keratinocyte λgt11 cDNA library with the PV IgG eluted from a 75 kD band that stained epidermis in pemphigus-like intercellular pattern and caused acantholysis in the keratinocyte monolayers revealed a novel type of AChRs, termed pemphaxin (a.k.a. annexin 9) [62]. Apparently, the AChR-binding pemphaxin is one of the annexin protein family members targeted by pemphigus autoimmunity [63]. Most recently, it has been reported that patients with endemic form of PF have autoantibodies to pilosebaceous units and to their surrounding neuro-vascular packages [64].

A large spectrum of organ-non-specific antigens that can be targeted by pemphigus autoimmunity (Table I) has been recently reviewed [39,65,66]. Although contribution of organ-non-specific antigens to keratinocyte detachment and death in pemphigus remains to be elucidated, it is plausible to speculate that some of them, e.g. tumor necrosis factor (TNF) receptor superfamily member 5, are involved in the activation of the extrinsic apoptotic pathway and others, e.g. NADH dehydrogenase-like protein, in the activation of the intrinsic pathway [37]. Of particular interest is a very high intensity of reactivity of PV IgG with Fc-IgG_2_ [49]. The fact that it has >95% homology with Fc-IgG_1_ may explain a hitherto mysterious ability of the chimeric baculoproteins containing the constant region of human IgG_1_ and Dsg 1 or Dsg 3, in contrast to the extracellular portion of Dsg 3 alone, to absorb out all disease-causing antibodies [67-69]. Thus, it can be concluded that pemphigus autoimmunity is directed against multiple organ-specific and non-organ-specific proteins, some of which are also targeted in other types of autoimmune diseases.

## Pemphigus autoantibodies

Exploration of novel self-antigens reacting with pemphigus antibodies remains one of the top priorities in pemphigus research, because binding of patients’ IgGs to these antigens triggers keratinocyte detachment and death in PV and PF. Although the mechanism of blistering in patients’ skin and mucosa involves various factors, including cell-mediated cytotoxicity, proteolytic enzymes, and pro-inflammatory and pro-apoptotic cytokines, the principal role of anti-keratinocyte antibodies in the pathophysiology of autoimmune pemphigus has been well documented. The major lines of evidence are as follows: (1) occurrence of transient pemphigus-like skin lesions in neonates born by mothers with active pemphigus; (2) induction of pemphigus-like phenotype upon passive transfer of patients’ IgGs to neonatal mice; and (3) elimination of disease causing activity of patients’ IgG fraction due to absorption with antigenic constructs. Despite enormous efforts to single out an autoantibody responsible for either PV or PF, none of hitherto reported results provides compelling evidence in favor of the monopathogenic hypothesis of pemphigus immunopathology.

A first successful attempt to reproduce disease phenotype in animal model was reported by Peterson and Wuepper [27] who observed small vesicles and limited areas of suprabasal acantholysis in the skin of neonatal mouse 36 h after intraperitoneal injection of a very high (40 mg) dose of rabbit IgG antibody raised against the 66 kD pemphigus antigen. Unfortunately, that antibody was not affinity purified on the antigenic peptide nor was its reactivity with keratinocyte proteins characterized. Some microscopic blisters also could be induced by pemphigus IgG eluted from a recombinant amino-terminus of Dsg 3 [67]. That IgG fraction uniquely recognized a 130kD protein of SDS-PAGE-resolved keratinocyte proteins. Although the anti-Dsg 3 antibody did not cause gross skin blisters, the obtained phenotype was deemed significant and the antibody termed “pathogenic". Paradoxically, gross blisters in that study were induced, albeit by the pemphigus IgG fraction depleted of Dsg 3 antibody, indicating that non-Dsg 3 antibodies were actually pathogenic. This finding, however, was interpreted as evidence that disease-causing antibodies are directed to other “conformational” epitopes of Dsg 3 [67].

Next, it was hypothesized that the extracellular domain of Dsg 3 should be combined with the Fc-portion of human IgG_1_ to create a proper conformation of the disease-specific Dsg 3 antigen [68]. Indeed, the obtained chimeric protein absorbed out all disease-causing antibodies and injection of preabsorbed PV IgG fraction to neonatal mice did not produce extensive skin blisters. Furthermore, the modified chimera that contained His residues attached to the Dsg3-Ig construct eliminated disease-causing antibodies from sera of patients with PAMS, and the eluted antibodies caused gross skin blisters in neonatal mice [70]. That same strategy was applied to create a “proper” conformational epitope of the Dsg 1 antigen capable of eliminating the disease-causing antibodies from PF sera [69]. Surprisingly, the antibodies eluted from neither chimeric construct were examined for their reactivities against keratinocyte proteins to confirm their unique specificity for the 130 kD Dsg 3 and 160 kD Dsg 1 antigens.

**Figure 3:**
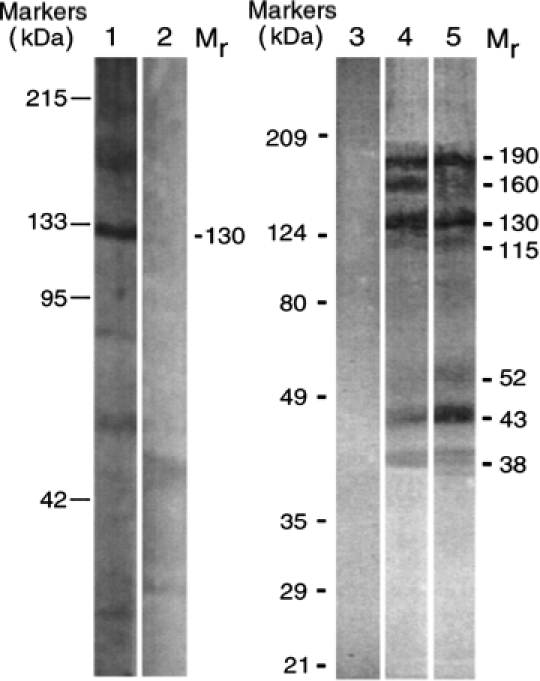
Profiles of PV IgGs absorbed by recombinant Dsg 1 and Dsg 3 baculoproteins in the Western blots of keratinocyte proteins resolved by 7.5% SDS-PAGE. Lane 1, reaction of PV IgG purified on the rDsg3-His construct with human keratinocytes. Lane 2, no primary antibody control for lane 1. Lane 3, no primary antibody control for lanes 4 and 5. Lane 4, reaction of PV IgG purified on the rDsgl-Ig-His construct with *Dsg3^null^* keratinocytes. Lane 5, reaction of PV IgG purified on the rDsg3-Ig-His construct with *Dsg3^null^”* keratinocytes. The positions of relative molecular mass (M_r_) markers run in parallel lanes of each blot are shown to the left of the respective blot. The apparent relative M_r_ of keratinocyte protein bands visualized due to PV antibody binding is shown to the right of lanes 2 and 5 in the columns designated M_r_. Modified from Ref. [18].

Successive studies demonstrated that the results obtained in experiments with recombinant (r)Dsg1-Ig-His and rDsg3-Ig-His chimeras that led to a conclusion that anti-Dsg 1 and anti-Dsg 3 antibodies are pathogenic in PF and PV, respectively, were complicated by the presence of non-Dsg antibodies [18]. Figure 3 shows that antibodies eluted from the chimeric constructs react with a mixture of keratinocyte proteins, including a non-Dsg 3 130 kD protein produced in the *Dsg3* keratinocytes used as a source of antigens. Noteworthy, the fact that the rDsg3-His construct was recognized by PV antibodies indicated that it actually had a proper confirmation. In contrast, the Fc-containing Dsg constructs evidently did not have proper confirmation.

These constructs were ill designed, because the ability of Fc fragment to mediate the Fc-Fc interactions known as Fc-mediated immune precipitation [71,72] was ignored. It has been recently demonstrated that the CH_2_ and CH_3_ domain regions of the Fc fragment used by Amagai et al. [68-70] in their pathogenic antibody elimination experiments provide an interface for the antigen-unspecific binding with another IgG molecule in immune complexes [73]. Thus, an antigen-unspecific, Fc-mediated immune precipitation can explain adsorption of multiple antibodies on the chimeric Dsg baculoproteins. However, one of the antibodies evidently was absorbed specifically. A protein array has revealed that PV patients produce anti-Fc-IgG antibody [49].

Several attempts have been made to produce a PV phenotype in mice by eliciting an innate anti-Dsg 3 antibody production. Immunization with recombinant Dsg 3, however, failed to induce acantholytic lesions despite explicit antibody production [74-76]. On the other hand, the results of experiments with *Rag2^−'−^* mice transplanted with the Dsg 3 antibody producing splenocytes from either rDsg3-immunized [77] or naive *Dsg3^−'−^* mice [78] suggested the pathogenicity of Dsg 3 antibody in PV. Since the recipient mice produced limited oral acantholysis and erosions, this mouse model was termed “active disease” [79]. A recent report, however, indicate that in the *Dsg3* splenocyte adoptive transfer model, the efficacy of anti-Dsg 3 antibody in causing acantholysis is <20% [80]. Such low frequency of functional anti-Dsg 3 antibodies observed in the recipient *Rag2∼* mice is consistent with inability of the majority of monoclonal murine [81] and human [82,83] anti-Dsg 3 antibodies to alter keratinocyte adhesion. Some anti-Dsg 3 antibodies, however, do cause acantholysis, albeit morphologically different from that found in PV patient's skin.

Anti-Dsg 3 antibody produces a desmosomal split without keratin retraction, apparently due to steric hindrance of Dsg 3 from opposing cells [84,85]. In marked contrast, numerous classical [86-89] and contemporary [55,90] electron microscopic studies of PV patients’ skin vividly demonstrated that desmosomes remain intact till the late stages of acantholysis when they are cleaved behind the desmosomal plaque, due to shearing forces produced by collapsing cells, and float free in the intercellular space. Thus, although the alterations of keratinocyte adhesion in recipient *Rag2^−'−^* mice are different from those taking place in patient's skin, these mice are still useful for studying the regulation of adaptive immune response to Dsg 3.

Although anti-Dsg 1 and 3 antibodies, alone or in combination, are not exclusively responsible for triggering intraepidermal blistering in patient's skin, they have a diagnostic utility. The Dsg 1 and Dsg 3 ELISAs provide a simple and highly sensitive approach to confirm the initial diagnosis of autoimmune pemphigus and differentiate it from other blistering diseases. The true value of ELISA results for patient management and prognosis, however, remains uncertain. The Dsg 1 and Dsg 3 IgG antibody titers do not always correlate with pemphigus disease activity [91,92] nor do they predict exacerbation and relapse of the disease [93]. Dsg 3 antibody can be absent in PV patients with active disease and present during remission [94-96]. Furthermore, anti-Dsg 1 or 3 antibodies have been detected in healthy subjects, relatives of pemphigus patients, and patients with irrelevant medical conditions [97-109].

Early reports suggested that levels of the IgG_4_ subclass of Dsg 3 antibody are associated with active disease and the IgG_1_ levels with remission [110-112]. The enthusiasm about using the IgG_4_ autoantibody titer for management of the disease, however, was dampened by results of a recent study. Although in patients with active pemphigus, IgG_4_ and IgG_1_ were the dominant subclasses (96% and 76%, respectively), in clinical remission the autoantibodies predominantly belonged to the IgG_2_ (75%) and IgG_4_ (37.5%) subclass. Circulating IgG_2_ and IgG_4_ subclass autoantibodies were also observed in 60% and 23.3%, respectively, of healthy relatives [101]. Additionally, some PV patients develop IgA and IgE classes of Dsg 3 antibodies [113,114].

It was originally thought that the clinical phenotype of pemphigus is defined by the anti-Dsg autoantibody profile as follows: anti-Dsg 1 antibody alone is associated with PF, anti-Dsg 3 antibody alone—with mucosal variant of PV, and both antibodies—with mucocutaneous variant of PV [115]. Although Dsg 1 antibody indeed appears to be a reliable serologic marker of PF and Dsg 3 antibody—that of PV, up to 58% of PF patients and 12% of patients with endemic PF (Fogo Selvagem) were reported to develop antibodies against both Dsg 1 and Dsg 3 [108,116,117]. Furthermore, it has been conclusively demonstrated that Dsg 1 and Dsg 3 testing cannot differentiate between various morphologic subtypes of PV [96,118,119]. In one study, for instance, 46% of PV patients did not have the PV phenotype (mucosal or mucocutaneous) predicted by their Dsg antibody profile [118]. In another study, the Dsg 1^+^/Dsg 3^+^ pattern was observed in 15% of PV patients with exclusive mucous membrane involvement [96].

A recent observation that an increase in Dsg 1 antibody titer in a PV patient has occurred already after the patient had started treatment and went into clinical remission [120] supports the notion that anti-Dsg antibodies “witness” rather than trigger PV, i.e. that production of these autoantibodies is the result rather than the cause of epidermal blistering in pemphigus [121]. Reactivity of pemphigus autoantibodies with both extracellular and intracellular domains of Dsg 1 and Dsg 3 [122] suggests that these antibodies are produced already after the whole Dsg molecules have been released from the CM of damaged keratinocytes into the intercellular space and became available to antigen-presenting cells. The presence of the N-terminal portion of Dsg 3 in human sera [123] lends additional support for the Dsg sloughing hypothesis.

Perhaps other desmosomal cadherins are also shed from the CM of damaged keratinocytes. The presence of anti-Dsc 1-3 antibodies in PV patients has been discovered relatively long ago [124,125], but the interest to these antibodies was diminished by the reports that none of 45 [126] or 74 [127] PV patients tested by ELISA had any anti-Dsc antibodies. However, a recent study showing that the Dsc 3 loss of function model exhibits a phenocopy of PV [43] suggested that anti-Dsc 3 antibody contributes to PV. As expected, the monoclonal antibody raised against the extracellular domain of Dsc 3 caused intraepidermal blistering in an *in vitro* model of human skin and a loss of cell-cell adhesion in the keratinocyte culture [128].

In the most recent study, 6 out of 38 PV and 1 out of 85 normal serum samples immunoprecipitated Dsc 3 [129]. Furthermore, while incubation of patient's IgG with human keratinocytes caused the loss of intercellular adhesion, adsorption with rDsc 3 prevented this effect [129]. Thus, while antibodies to the desmosomal cadherins may be playing a scavenging role by eliminating CM debris from the intercellular spaces of damaged epidermis they may obstruct homophilic and heterophilic binding between the neighboring keratinocytes, thus contributing to acantholysis.

The initial insult that triggers keratinocyte damage in pemphigus is apparently sustained by autoantibodies to the cell membrane receptors whose ligation causes cell shrinkage—the earliest sign of keratinocyte detachment in pemphigus lesions [55, 86-90] that leads to ripping desmosomes off the CM. Indeed, it has been demonstrated that rabbit anti-a9 AChR antibody causes keratinocyte shrinkage and rounding up [36]. Noteworthy, pemphigus IgG produces similar morphologic changes in keratinocyte monolayers [130].

Likewise, pharmacologic inhibition of a3 nAChR causes keratinocytes to retract their cytoplasmic aprons, shrink, and round up [131]. Pemphigus-like acantholysis has also been observed due to inhibition of keratinocyte mAChRs [130], in keeping with the synergistic control of keratinocyte adhesion by auto/paracrine ACh that maintains the polygonal shape of keratinocytes and their adhesion by simultaneously activating both classes of keratinocyte cholinergic receptors [132,133]. Hence, blockade of any type of keratinocyte AChRs by pemphigus IgGs can trigger acantholysis.

Although the hypothesis of primary involvement of autoantibodies against canonical AChR subtypes in pemphigus pathophysiology is awaiting its *in vivo* confirmation, the already completed studies have demonstrated essential role of antibody against the non-canonical ligand of AChRs termed pemphaxin [62]. Preabsorption of PV sera with recombinant pemphaxin eliminated acantholytic activity and eluted antibody immunoprecipitated native pemphaxin. Although anti-pemphaxin antibody alone did not cause skin blisters *in vivo,* its addition to the preabsorbed PV IgG fraction restored the acantholytic activity of passively transferred antibodies [62].

These observations indicate that pemphaxin is an essential part of the pool of keratinocyte cell surface antigens that should be simultaneously targeted by autoantibodies to induce acantholysis. The anti-mitochondrial antibodies from different PV patients that recognized distinct combinations of antigens with apparent MWs of 25, 30, 35, 57, 60, and 100 kD evidently are also pathogenic, because their absorption abolished the ability of PV IgG to cause keratinocyte detachment both *in vitro* and *in vivo* [37].

Involvement of multiple autoantibody specificities in pemphigus pathogenesis is explained through the “multiple hit” hypothesis [134] as follows: anti-AChR antibodies trigger acantholysis by weakening cohesion of neighboring keratinocytes due to inhibition of the physiologic control of their polygonal shape and intercellular attachment. The affected keratinocytes shrink, causing desmosomes to be sloughed in the intercellular space. The adhesion molecules floating free in the intercellular space bring about a reciprocal production of scavenger antibodies that, in turn, saturate epidermis, thus preventing nascent desmosome formation by steric hindrance. Thus, according to the multiple hit hypothesis, pemphigus results from a synergistic and cumulative effects of autoantibodies targeting keratinocyte CM antigens of different kinds including (i) molecules that regulate cell shape and adhesion (e.g. AChRs); and (ii) molecules that mediate cell-cell adhesion (e.g. desmosomal cadherins). Severity of the disease and exact clinical picture depend on the ratio of different kinds of autoantibodies in each particular patient.

**Figure 4:**
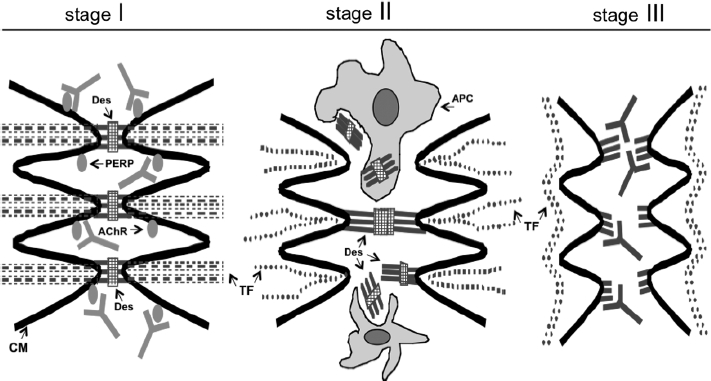
Hypothetical scheme of the time course of pathobiologic events leading to acantholysis in pemphigus. In stage I, antibodies to PERP and/or cellular AChRblock the physiologic control of polygonal cell shape and intercellular adhesion. This increases phosphorylation of adhesion molecules with their subsequent dissociation from the adhesion units on CM, and also initiates programed cell death. In stage II, the tonofilament (TF). cytoskeleton collapses and keratinocytes shrink with associated sloughing of desmosomes which elicits autoimmune response to the desmosomal antigens. In stage III, anti-Dsg antibodies bind to their targets on the CM of keratinocytes thus precluding formation of new intercellular junctions. Modified from Ref. [55].

In conclusion, different patients develop distinct constellations of autoantibodies which, together with the individual's re-epithelialization abilities, determine clinical severity of disease, its natural course, and response to treatment. The Dsg 1 and 3 antibodies are the sensitive markers of pemphigus, but their primary role in the pathogenesis of PF and PV, respectively, is overestimated. Therefore, not surprisingly, clinical trial of the Dsg 3 peptides (PI-0824 vaccine) has not shown the anticipated clinical or immunologic activity (reviewed in [135]). Apparently, an attack by a constellation of autoantibodies simultaneously targeting several keratinocyte proteins is required to disrupt the integrity of epidermis. The multiple hit hypothesis reconciles findings of anti-AChR autoimmunity with the fact that pemphigus patients also develop autoantibodies to adhesion molecules as well as various other proteins. The hypothetical sequence of immunopathologic and biochemical events leading to acantholysis in pemphigus is shown in Figure 4. Future studies should define the autoantibodies that sustain an initial insult triggering keratinocyte detachment and those produced as a result of primary cell damage to clean up the proteins released in the intercellular spaces by damaged cells.

## Regulation of pemphigus autoimmunity

Although autoimmunity is a normal event, autoimmune diseases result from an aberration of the normal phenomenon [136]. The etiology of this switch in pemphigus is apparently multifactorial, with the final common pathway being a loss of normal self-tolerance in the stratified squamous epithelium. Analysis of genetic factors contributing to PV and PF has shown that the same genetic regions can contribute to both forms of the disease (reviewed in [137]). The HLA genes are probably the most significant genetic predisposition factors, because they play an important role in the antigen presentation process, whereas other loci may participate in an additive or epistastic manner.

Population studies have consistently shown a link between certain class II HLA alleles and distinct ethnic groups of pemphigus patients. A recent study involving a large cohort of White European and Indo-Asian patients with PV confirmed associations with the alleles HLA DRβ1*0402 and 1404, and DQβ1*0302 and 0503 [138]. The DRβ1*1404 was the strongest risk factor in the Indo-Asian group and DRβ1*0402—in the White European group. In White Europeans, a significant association was also shown for the novel allele DRβ1*1454 [138]. Also, it has been documented that HLA-DRβ1*0402 is associated with PV in Jewish and HLA-DQ(31* 0503 in non-Jewish populations [139-141].

Although HLA studies have shown that susceptibility to PF also correlates with the presence of DR4, DR14, and DR1 alleles, in contrast to PV, no single DR4 or DR14 allele was found to be overrepresented in PF patients [137]. The HLA-DR/DQ distributions does not differ among PV patients according to the presence or absence of anti-Dsg 1 coexisting with anti-Dsg 3 [142].

Although the basis for autoimmunity in pemphigus remains unrecognized, the regulation of anti-keratinocyte autoimmunity in PV and PF has been studied based on the assumption that Dsg 1/3 antibodies solely represent pemphigus autoimmunity. It was reported that the Th1 and Th2 cell recognition of Dsg 3 peptides is restricted by HLA-DRβ1*0402 and/or HLA-DQβ1*0503, and that the proliferative response of autoreactive Th cells can be blocked by anti-DR and anti-DQ antibodies, respectively [143-146].

A loss of self-tolerance against Dsg 3 in both T and B lymphocytes was found to be required for efficient production of anti-Dsg 3 IgG antibodies [147-149]. The anti-Dsg 3 antibody production in mice was inhibited by the anti-CD 154 monoclonal antibody that blocks CD40L-CD40 interaction [150]. The CD8^+^ T cells specific for Dsg 3 were also detected in PV patients [151], which is in keeping with earlier observation of the autoreactive cytotoxic T lymphocytes are sensitized to putative keratinocyte antigens in PV patients [152].

Autoimmunity to certain epitopes of Dsg 3 may be a normal event, because Dsg 3-reactive B cells as well as Th1 and Th2 cells are present in normal individuals [97,100,101,146,151,153,154]. The presence of autoreactive B cells is evidenced by production of anti-Dsg 3 antibodies by healthy relatives of PV patients [97,100,101,154]. The presence of Dsg 3-reactive Th1 cells has been demonstrated in healthy carriers of PV-associated HLA class II alleles, and the Dsg 3-reactive Th1 clones derived from these individuals were restricted by HLA-DRβ1*0402 and DQβ1*0503 [146,151,153].

There is a predominance of autoreactive Dsg 3-reactive Th1 cells in healthy individuals and Dsg 3-reactive Th2 cells in PV patients [146]. However, Dsg 3-reactive Th2 cells are detected at similar frequencies in acute onset, chronic active, and remittent PV, while the number of autoreactive Th1 cells exceeds that of Th2 cells in chronic active PV [146]. Likewise, Dsg 1-responsive Th1 and Th2 cells were also found both in patients with PF and in healthy individuals [155].

Defects in Tregs have been reported in a wide variety of human organ-specific autoimmune diseases [156]. While the induction of Treg suppressive activity is specific and requires antigenic stimulation through T-cell receptor, the suppression exerted by Tregs is antigen nonspecific [157]. Various Tregs can employ distinct mechanisms to collaboratively regulate the duration and magnitude of an immune response [158]. It has been postulated that active immune suppression operates in healthy individuals possessing Dsg 1- and Dsg 3-reactive T cells, and that an imbalance of the putative relationship between autoreactive Th and Tregs (Tr1) cells is critical to the development of pemphigus [159]. In support, the CD4^+^CD25 ^+ hi^ Tregs are decreased in peripheral blood of PV patients [160]. Furthermore, a subset of Dsg 3-reactive, interleukin (IL)-10-secreting Tr1 cells was found in the majority of healthy carriers of PV-associated HLA class II alleles and only in <20% of PV patients [161]. However, a recent study demonstrated that PV skin lesions contain both Foxp3-expressing cells and IL-17 producing CD4^+^ cells [162].

Thus, deficiency of Tregs in PV patient's blood is not accompanied by a decrease in Tregs in PV lesions, and a decrease in Tregs in peripheral blood may result from accumulation of Tregs in skin lesions and draining lymph nodes [162]. Alternatively, or additionally, pemphigus autoimmunity may be triggered via the pathway involving activation of toll-like receptors (TLRs). This mechanism has been suggested by a recent demonstration of reversible relapse of PF triggered by the TLR7 agonist imiquimod [163]. In this scenario, B-cell tolerance is broken due to ligation of B-cell receptor and TLR by self-antigen/TLR ligand, leading to breakage of T-cell tolerance and activation of autoreactive B and T cells [164].

**Figure 5:**
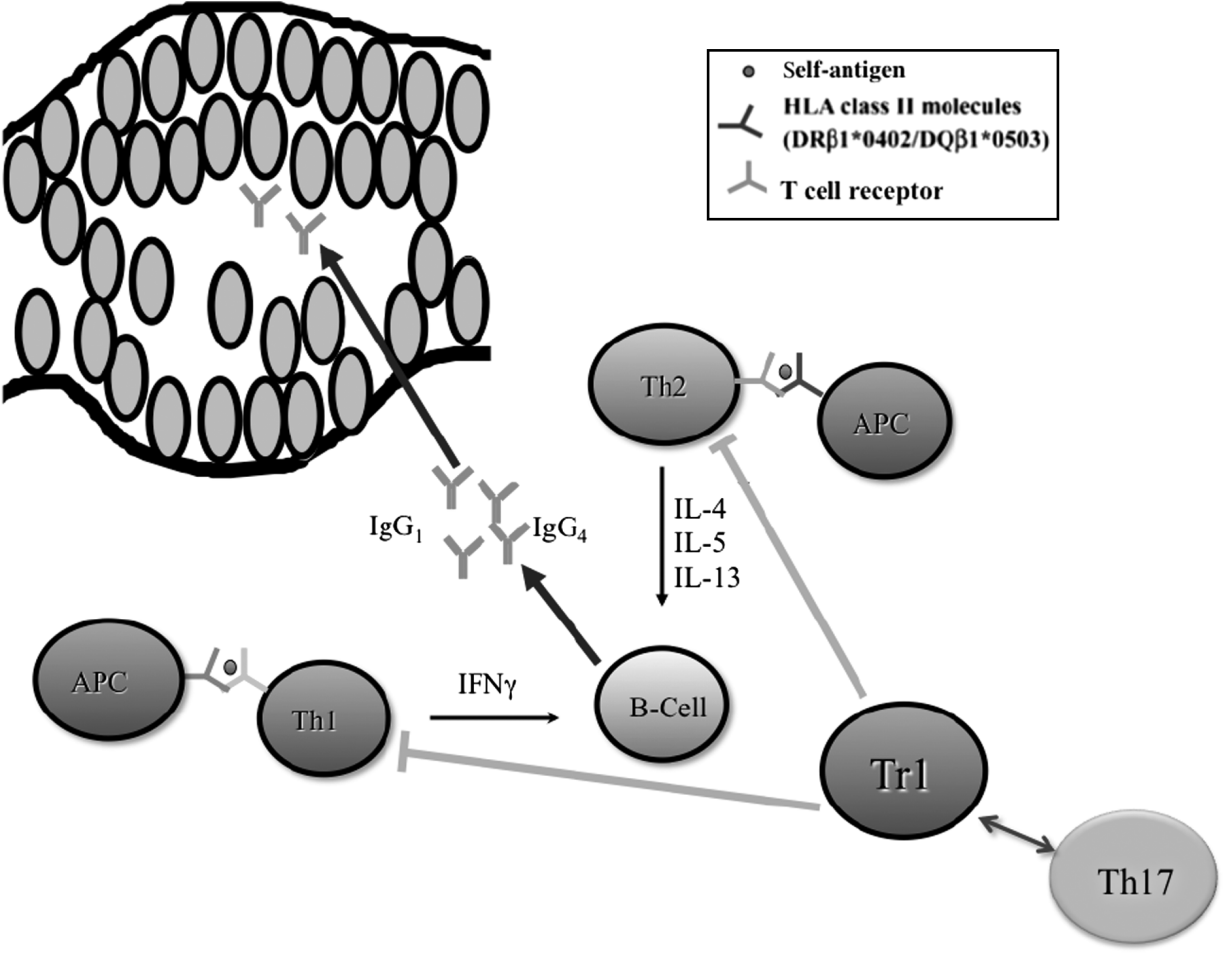
Hypothetical scheme of immune dysregulation in PV. *Abbreviations:* APC, antigen-presenting cells; IL, interleukin. Modified from Ref. [339].

In conclusion, regulation of Dsg 1 and 3 antibody production agrees with the basic postulates of fundamental immunology on T cell-B cell cooperation. Th1 and Th2 cells found in patients with PV and healthy carriers of PV-associated HLA class II alleles recognize identical epitopes of the Dsg 3 ectodomain presented by antigen-presenting cells. A decrease in Tregs in peripheral blood of PV patients does not validate the postulated deficiency of the immunosuppressive activity, because Tregs are present in PV lesions. Future studies of the immunoregulatory mechanisms of PV should characterize the reputed interplay between Tregs and Th17 cells (Figure 5), and identify the role for TLRs that can regulate function of Th1, Th2, Th17 cells, and Tregs.

## Molecular mechanisms of keratinocyte detachment in pemphigus

Several hypotheses have been put forward to explain the mechanism of pemphigus acantholysis. The classical explanation through the hypothesis of steric hindrance of Dsg 1- and Dsg 3-mediated adhesion by respective antibodies [165,166] has been challenged by numerous reports showing activation of specific signaling pathways in keratinocytes exposed to pemphigus IgGs. The steric hindrance hypothesis is based on the erroneous assumptions that binding of PV IgG in the epidermis is limited to desmosomes [167] and that the phenocopies of PF and PV in mouse skin can be induced by passive transfer of PV IgGs recognizing uniquely the desmosomal cadherins Dsg 1 and 3, respectively [68,69].

In fact, although Dsg molecules are indeed predominantly localized to desmosomes [168], binding of PV IgG extends well beyond the desmosomes decorating the entire surface of keratinocytes [169]. As already mentioned, the Dsg-Fc-IgG constructs were found to be not specific for Dsg 1 or 3 antibodies [18,36]. Furthermore, PF IgG has recently been shown to cause dissociation of keratinocytes without blocking Dsg 1 homophilic transinteraction [170]. Surprisingly, this cumulative evidence that non-Dsg molecules are targeted by pathogenic PV IgG in the interdesmosomal areas is being interpreted as targeting of Dsg 3 located outside of the desmosomes [171].

The alternative Dsg 3 alteration hypotheses proposed by different authors [171-173] have one common theme. They are based on the assumption that all outside-in signals elicited due to binding of PV IgG to keratinocytes emanate exclusively from Dsg 3. To account for a very broad spectrum of signals, it was inferred that Dsg 3 can act as both adhesion receptor and signal transmitter [171-173]. The following hypothetical chain of events was envisioned: (1) PV IgG signaling is initiated by ligation of nonfunctional pool of Dsg 3 outside of the desmosome; (2) when ligated by an autoantibody, these putative nonfunctional Dsg 3 molecules, either membrane associated or internalized or both, send signals that impair Dsg 3 trafficking into and out of desmosome; and (3) the supposedly impaired Dsg trafficking specifically depletes Dsg 3 from desmosomes without changes in other functional proteins.

The notion about principal role of Dsg 3 in PV IgG signaling stems from experiments in which the whole PV IgG fraction, rather than affinity purified patient's anti-Dsg 3 antibody, was used to elicit biologic responses [174-183]. When interpreting results, it was assumed that the whole plethora of PV IgG effects, including Dsg 3 endocytosis and activation of various signaling cascades, resulted exclusively from Dsg 3 ligation by an autoantibody. However, Jennings et al. [184] have recently demonstrated an explicit biologic effect of PV IgG on the keratinocytes expressing the Dsg 3 molecules whose “pathogenic” epitopes were hidden due to cell preincubation at 4°C with the anti-Dsg 3 monoclonal antibody AK23 that reportedly reproduces PV phenotype [81,185] and induces PV IgG-like signaling [186].

Therefore, depletion of Dsg 3 is apparently a secondary event resulting from an inside-out signaling caused by keratinocyte response to the pathogenic autoantibodies that deliver an initial insult. The primary role of anti-PEPR antibody in the depletion of Dsg 3 was suggested by an observation that binding of PV antibodies triggers internalization of PERP, which enhances depletion of desmosomal Dsg 3 and intercellular adhesion defects [187]. Thus, endocytosis of the immune complexes containing non-junctional Dsg 3 and depletion of Dsg 3 from desmosomes apparently represent two independent events, with the former being a natural outcome of formation of antigen-antibody complex on the CM and the latter resulting from the inside-out signaling altering function and trafficking of desmosomal cadherins.

The outside-in signaling elicited due to binding of PV IgGs to keratinocytes proceeds via different pathways, consistent with simultaneous ligation of several types of cell surface receptors by distinct antibodies produced by pemphigus patients. Different research groups reported engagement of Src, epidermal growth factor receptor (EGFR) kinase (EGFRK), cAMP, protein kinases A and C (PKC), phospholipase C, mTOR, p38 MAPK, JNK, other tyrosine kinases, and calmodulin [37,47,175,188-191]. The preferential signaling pathway downstream of targeted self-antigens is apparently determined by a unique composition of the pool of anti-keratinocyte antibodies produced by each PV patient, because IgG fractions from different PV patients exhibit distinctive time patterns of kinase activation [37].

Timing of kinase activation is critical for understanding the hierarchy of signaling events leading to acantholysis. The time course studies demonstrated that the activities of Src and EGFRK peak at 30-60 min after exposure to PV IgG [37,47], suggesting that engagement of Src/EGFRK is a key step that relays signals emanating from ligated antigens to the intracellular effectors affecting keratinocyte adhesion and viability.

Activation of EGFRK due to binding of PV IgG to keratinocytes is followed by phosphorylation of its downstream substrates, the MAP kinase ERK and the transcription factor c-Jun [189]. Activation of PKC is also one of the earliest events in PV IgG-induced acantholysis [192]. The elevation of p38 MAPK activity caused by antibodies from some PV patients can be observed already at 15 min, while the majority of PV IgGs activate p38 MAPK after a prolonged incubation [37]. Thus, it is becoming evident that an array of interconnected signaling cascades emanating from different cell surface antigens simultaneously targeted by a constellation of patients’ anti-keratinocyte antibodies trigger acantholysis and keratinocyte death in pemphigus.

Two different approaches have been used to elucidate involvement of Dsg 3 in the PV IgG-mediated signaling and define relevant pathways. Through one approach, the Dsg1 or Dsg3 genes in cultured human keratinocytes were silenced using the RNA interference technology [47]. Transfection with small interfering RNAs that inhibited expression of either Dsg 1 or Dsg 3 or both in all cases blocked approximately 50% of p38 MAPK activity, but only slightly altered the PV IgG-dependent raise in Src and EGFRK activities. To avoid any possible contribution to PV IgG signaling by residual Dsg 3 protein, a separate series of experiments employed keratinocytes grown from the epidermis of neonatal Dsg 3 knockout mice [37]. It was documented that lack of Dsg 3 did not affect the ability of PV IgG to activate Src and EGFRK.

However, in the absence of Dsg 3, the PV IgG-dependent activation of both p38 MAPK and JNK was significantly reduced. Because both p38 MAPK and JNK can be activated secondary to keratinocyte shrinkage and detachment [193-195], and since keratinocyte damage in PV is associated with activation of the cell death program (reviewed in [196]), it was important to determine whether p38 MAPK and JNK activation precedes or follows launching of the apoptotic cascade. Inhibitors of the executioner caspases abolished activation of JNK and the late p38 MAPK peak [37], indicating that these activities were indeed triggered by the cell injury rather than by the PV IgG binding to keratinocyte antigens. This supposition has been recently corroborated by a report that p38 MAPK activation occurs downstream at the loss of intercellular adhesion in PV [197].

Thus, although the pool of anti-keratinocyte antibodies produced by PV patients contains anti-Dsg 1 and/or 3 antibodies, published studies indicate that non-Dsg antibodies are the major contributors to early signaling events. Early activation of the Src/EGFRK and PKC-dependent pathways is apparently pathogenic because it leads to acantholysis [47,189], late activation of p38 MAPK is secondary to cell detachment [197], whereas activation of the cAMP/protein kinase A step appears to have a protective function [191].

The principal signaling event leading to acantholysis is triggered due to antibody interference with the physiologic control of keratinocyte survival, shape, and adhesion. For instance, blocking of keratinocyte AChRs interferes with auto/paracrine control of assembly/disassembly of intercellular junctions, cell shape, motility, proliferation, apoptosis, and differentiation (reviewed in [198]). Both muscarinic and nicotinic antagonists have been shown to widen the intercellular spaces in epidermis and cause overt acantholysis *in vitro* through the mechanism that may involve alterations of both production and phosphorylation of keratinocyte adhesion molecules (reviewed in [55]). It is well known that phosphorylation of adhesion molecules plays an important role in assembly/disassembly of intercellular junctions [199-203], and that phosphorylation of cadherin [204-206], γ-catenin [207], desmoplakin [208,209], and Dsg [210-212] is associated with a loss of adhesion.

Some intercellular junction proteins are phosphorylated on serine, some on tyrosine, and some on both residues. The seminal works by Aoyama et al. [212,213] demonstrated that binding of PV IgG to cell surface antigens induces phosphorylation of Dsg 3, its dissociation from plakoglobin, and formation of Dsg 3-depleted desmosomes. These findings were corroborated by the results showing that in addition to Dsg 3, PV IgG also increases the level of phosphorylation of keratinocyte E-cadherin as well as β-, γ7-, and p120 catenins [214,215]. These experiments also demonstrated that keratinocyte dyshesion correlates closely with the degree of tyrosine phosphorylation of p120-catenin by Src and serine phosphorylation of β-catenin by classic PKC isoforms [215].

**Figure 6:**
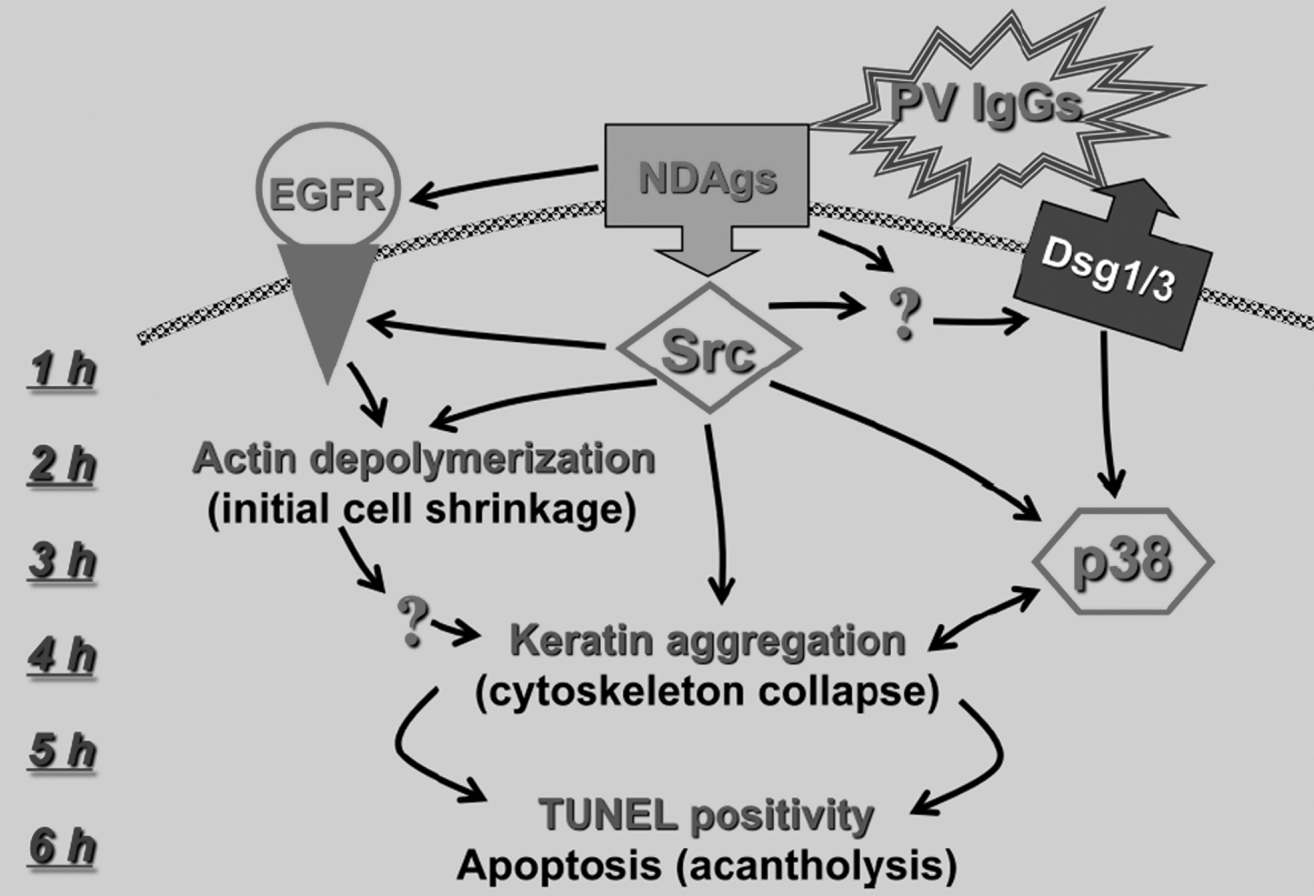
Hypothetical scheme of early signaling steps during first 6h after PV IgG binding to keratinocytes and their correlation with the major intracellular pathobiologic events. *Abbreviations:* EGFR, epidermal growth factor receptor; Dsg, desmoglein; NDAgs, non-Dsg antigens. From Ref. [47].

The Src-dependent cascade is also responsible for keratinocyte shrinkage (cell volume reduction) and keratin aggregation [47]. The cytoskeletal collapse has been reported as an early event in pemphigus acantholysis that precedes visible separation of keratinocytes [47,179,216-218]. Thus, it appears that PV IgG-induced phosphorylation of adhesion molecules and structural proteins leads to weakening of intercellular junctions and collapse of the cytoskeleton, respectively. The chronological scheme of early signaling and pathobiologic events in keratinocytes exposed to PV IgG are shown in Figure 6.

Numerous classical and modern clinical and experimental studies in pemphigus demonstrated that desmosomes separate when the intercellular spaces are already widened [87,90,219-222]. Desmosomes do not split and disappear until late in acantholysis when keratinocytes are almost completely separated from each other. At early stages of acantholysis, the half-desmosomes remain invisible because they are firmly adhering to each other. In late acantholysis, some half-desmosomes adhere to each other so strongly that they can be ripped off one cell while remaining adherent to their counterpart on the opposite cells.

Hence, disruption of intercellular bridges results from ripping intact desmosomes off the plasma membrane of collapsing keratinocytes by shearing forces. The intact desmosomes ripped off from neighboring cells can be seen floating free in the intercellular space, which is in keeping with the sloughing of desmosomal cadherins that gives rise to scavenging autoantibodies. The “basal cell shrinkage” hypothesis reconciles the time course of acantholysis in PV with the characteristic appearance of acantholytic epidermis, known as “tombstoning” [223]. According to this hypothesis: (1) keratinocytes separate because they shrink more than can be held together by desmosomes; (2) the suprabasal clefting occurs because basal cells shrink more than suprabasal keratinocytes; and (3) pharmacologic inhibition of the principal signaling pathways leading to cytoskeletal disorganization should prevent pemphigus acantholysis.

One of the most important recent advances in pemphigus research was elucidation of the molecular mechanisms that selectively target basal cells in PV, as predicted by the basal cell shrinkage hypothesis. Pretel et al. [190] demonstrated that pretreatment with the mTOR inhibitor sirolimus prevented suprabasal acantholysis in the epidermis of neonatal mice injected with PV IgG. In this model, PV antibodies caused unopposed upregulation of mTOR selectively in basal keratinocytes, which was associated with the appearance of signs of apoptosis that were also abolished by sirolimus [190].

Downstream of mTOR, the induction of keratinocyte apoptosis by PV antibodies apparently proceeds through the c-Myc-dependent pathway [218]. c-Myc-induced apoptosis involves caspase 9 [224], which is activated in keratinocytes treated with PV IgG [225]. In agreement with the fact that cyclin-dependent kinase 2 (Cdk2) is required by c-Myc to induce apoptosis [226], PV sera induces accumulation of Cdk2 that contributes to acantholysis in the mouse model of PV [227]. Indeed, sirolimus has been shown to inhibit expression of both c-Myc [228] and Cdk2 [229]. Taken together, these observations help explain why epidermal clefting in PV always occurs just above basal cells (tombstoning), despite deposition of IgG antibodies throughout the entire epidermis.

Both extrinsic and intrinsic pathways of cell death triggered in keratinocytes by PV IgGs can lead to the structural damage manifested by basal cell shrinkage. It has been documented by different research groups that in the skin of PV patients, keratinocytes exhibit signs of apoptosis that precede their detachment and blister formation, and that PV IgG and sera induce biomolecular markers of apoptosis and oncosis in keratinocyte monolayers and skin organ cultures [225,230-235]. Two groups of PV patients, each producing autoantibodies activating predominantly either apoptotic or oncotic cell death pathway, have been identified [225].

The anti-PERP PV antibody may launch the cell death pathways in keratinocytes, because PERP expression leads to activation of an extrinsic receptor-mediated apoptotic pathway with a possible subsequent engagement of the intrinsic apoptotic pathway [54]. Anti-mitochondrial PV antibodies also can trigger intrinsic apoptotic cascade in keratinocytes [37]. Other types of autoantibodies and soluble mediators of inflammation can activate cell death pathways in keratinocytes. Dr Pincelli's group demonstrated that Fas ligand (FasL) in pemphigus sera induces keratinocyte apoptosis through activation of caspase 8 [231], and that FasL neutralizing antibody prevents PV IgG-induced apoptosis both *in vitro* and *in vivo* [236].

Furthermore, it has been shown that TNFa mRNA is abundantly expressed in PV skin lesions [237,238]; serum TNFa levels correlate closely with disease activity and autoantibody titers [239,240]; and anti-TNFa antibody inhibits acantholysis induced by PV autoantibodies *in vitro* [237]. The synergistic acantholytic effects of PV IgG, FasL, and TNFa were documented in experiments with keratinocyte mono-layers and full thickness equivalents of human epidermis [241]. These mediators of apoptosis as well as the increased amounts of activated kallikreins [242] and several types of inflammatory cytokines [237,239,243,244] found to be elevated in pemphigus sera may account for a minor acantholytic activity of the PV serum depleted of IgG, as reported by Cirillo et al. [245].

Besides PV IgGs, cell death pathways in pemphigus can be triggered by autocrine and paracrine factors released from damaged keratinocytes *in situ*. PV IgG binding to keratinocytes not only elicits secretion of soluble FasL but also increases expression of Bax, Fas receptor (FasR), coaggregation of FasL and Fas R with caspase 8 in a membranal death-inducing signaling complex, and downregulation of the anti-apoptotic Bcl-2, FLIP-1, and the oncosis inhibitor calpastatin [189,225,231,232,234,246,247]. In turn, TNFa induces urokinase plasminogen activator mRNA [237].

**Figure 7:**
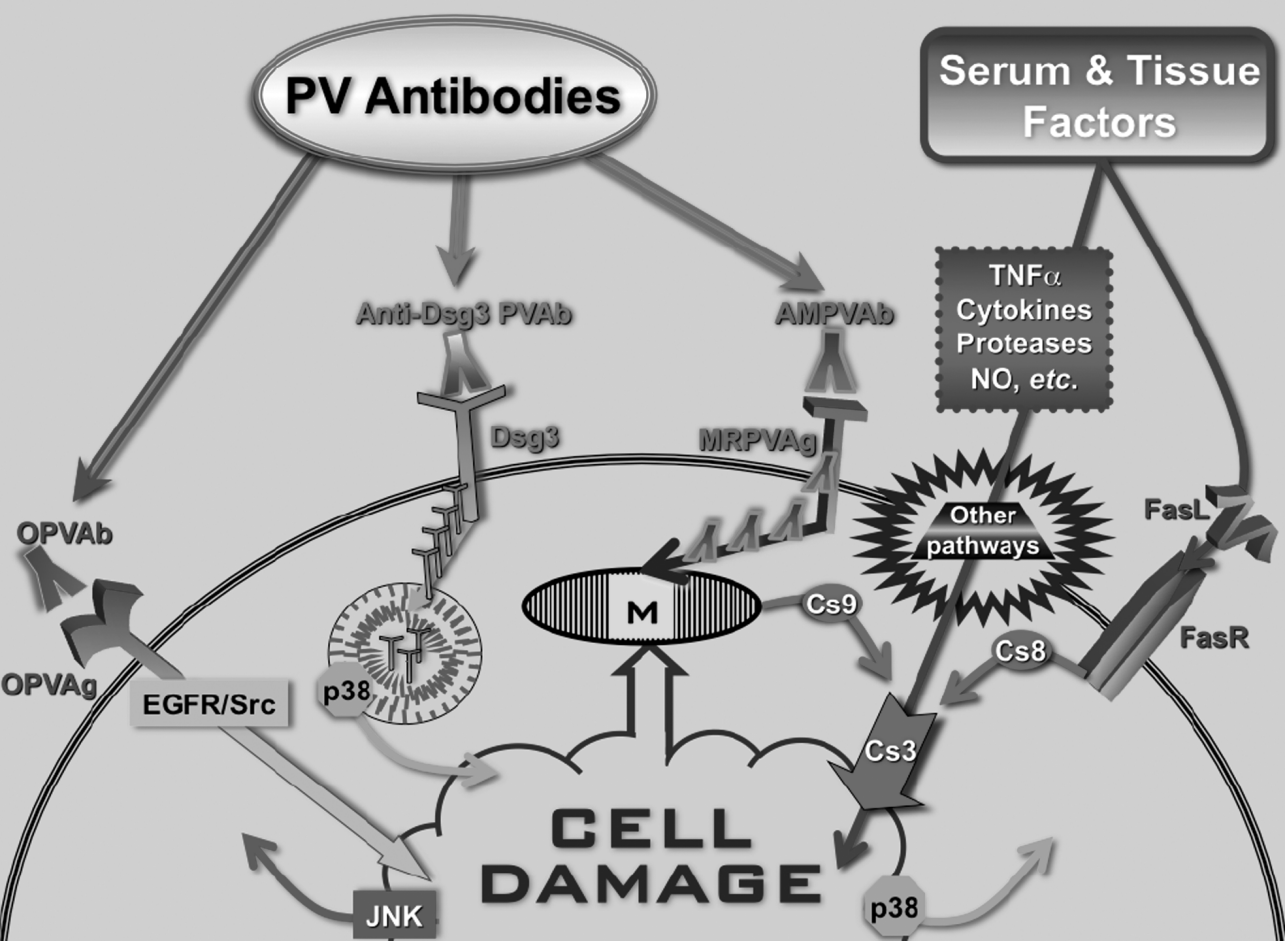
Hypothetical scheme of signaling events mediating keratinocyte damage in PV. *Abbreviations:* AMPVAb, anti-mitochondrial PV antibody; Cs, caspase; FasL, Fas ligand; FasR, Fas receptor; MRPVAg, mitochondria-related PV antigen; NO, nitric oxide; OPVAb, PV antibodies of other specificities, including anti-AChR and anti-PERP antibodies; OPVAg, other types of putative PV antigens; PVAb, PV antibody; TNFa, tumor necrosis factor *a*. Modified from Ref. [37].

It is well known that blister fluid and/or perilesional skin of PV patients contains high levels of proteases and various inflammatory cytokines that may contribute to acantholysis [242,248-250]. Thus, a simultaneous autoimmune attack by the three classes of autoantibodies against desmosomal, mitochondrial, and other keratinocyte autoantigens, such as AChRs and PERP, may be required to induce pathologic changes in patient's skin. The anti-keratinocyte antibodies synergize with the effectors of apoptotic pathway FasL and TNFa as well as proinflammatory/ cytotoxic serum and tissue factors, such as serine proteases and cytokines, that altogether overcome the natural resistance and activate cell death pathways in keratinocytes (Figure 7).

Acantholysis and cell death (apoptosis/oncosis) are inseparable in PV because both processes are triggered by the same signal effectors activated due to PV IgG binding to keratinocytes and mediated by the same set of cell death enzymes. This is evident from the reports that inhibitors of Src, EGFRK, p38 MAPK, and mTOR block both acantholysis and apoptosis [1,47,176,177,189,190,215,251] and caspase inhibitors prevent acantholysis both *in vitro* and *in vivo* [190,225,246]. Moreover, it has been demonstrated that apoptotic enzymes cleave Dsg 1, 2, and 3 [236,252,253].

**Figure 8:**
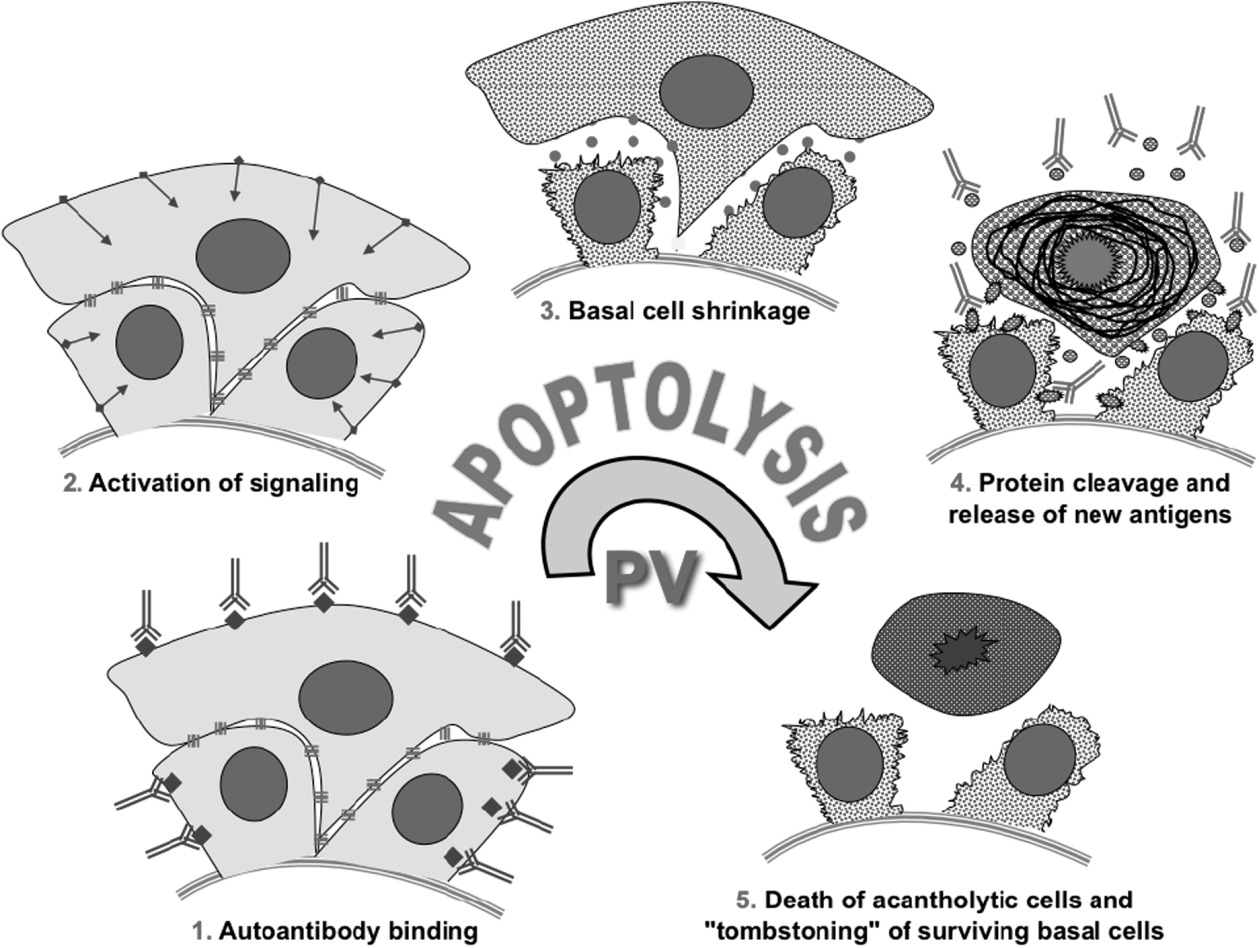
Hypothetical scheme of keratinocyte apoptolysis in PV. Step 1: apoptolysis is triggered by binding of autoantibodies to the PV antigens capable of transducing apopotolytic signals from the keratinocyte plasma membrane, such as PERP and AChRs. Step 2: outside-in signaling from ligated antigens launches the cell death cascades. Step 3: collapse and retraction of the TFs cleaved by executioner caspases and dissociation of interdesmosomal adhesion complexes caused by phosphorylation of adhesion molecules result in basal cell shrinkage, most of desmosomes remain intact. Step 4: massive cleavage of cellularproteins by activated cell death enzymes leads to collapse of the cytoskeleton and tearing off desmosomes from the CM with subsequent production of scavenging (i.e. secondary) autoantibodies mainly to sloughed adhesion molecules. Step 5: suprabasal acantholytic cells die rendering a tombstone appearance to surviving basal cells. Modified from Ref. [1].

For instance, when PV IgG was added to keratinocytes in the presence of anti-FasL neutralizing antibody, the cleavage of the intracellular portion of Dsg 3 and its degradation decreased [1]. The fact that structural damage and death of keratinocytes in PV are mediated by the same set of enzymes has justified introduction of the new term “apoptolysis” to distinguish the unique mechanism of autoantibody-induced keratinocyte damage in PV from other known forms of cell death [1]. The apoptolysis hypothesis links the basal cell shrinkage to suprabasal acantholysis and cell death, and emphasizes that apoptotic enzymes contribute to acantholysis in terms of both molecular events and chronologic sequence. It postulates that cell detachment and death in PV develop through the following five major steps (Figure 8):

- *Step 1:* binding of pathogenic antibodies to keratinocytes via a receptor-ligand type of interaction sends an array of the agonist- and antagonist-like signals;
- *Step 2:* activation of Src, EGFRK, p38 MAPK and mTOR, and other signaling elements downstream of ligated PV antigens and elevation of intracellular Ca^2+^, altogether, initiate cell death enzymatic cascades;
- *Step 3:* suprabasal acantholysis starts when basal cells shrink due to reorganization of cortical actin filaments, collapse and retraction of the tonofilaments (TFs) cleaved by executioner caspases, as well as dissociation and internalization of intercellular adhesion complexes caused by phosphorylation of adhesion molecules and their cleavage by caspases;
- *Step 4:* acantholysis advances due to continued degradation and massive collapse of structural proteins by the same cell death enzymes, leading to separation and rending of preexisting desmosomes from the CM by shear forces, thus separating the collapsing cells and stimulating production of secondary (scavenging) antibodies;
- *Step 5:* rounding up and death of acantholytic cells in the lower epidermal compartment follows irreversible damage of mitochondrial and nuclear proteins.

In conclusion, the mechanism of apoptolysis in PV encompasses several tiers of events triggered through distinct antigen-antibody systems. Because apoptolysis develops in a stepwise fashion, late morphologic features of apoptosis, such as rounding up, nuclear fragmentation, and plasma membrane blebbing, do not become apparent until the keratinocyte death in Step 5. Signaling mechanisms may vary from patient to patient because a unique composition of the pool of autoantibodies determines the principal pathway. The Dsg 3-dependent late peak of p38 MAPK activity and activation of JNK represent the pathways mediating processing and utilization of internalized Dsg 3, rather than a primary downstream signaling emanating from the CM. The apoptolysis hypothesis has several important implications: (1) it links together a number of apparently unrelated, and previously held contradictory, observations on the events surrounding acantholysis; (2) it opens new avenues of investigation into the pathomechanism of pemphigus; and (3) it creates new approaches to the treatment of pemphigus based on interfering with or blocking the signaling pathways and enzymatic processes that lead to blistering.

## Treatment of pemphigus

The high-dose, long-term systemic glucocorticoid therapy remains the mainstay of current therapy of PV and PF patients. Corticosteroid hormones (usually prednisone tablets) are essential to establish control of PV during the acute stage [2,254,255]. Optimal dosing proved to be variable and could not be predicted at the outset in any given patient [4]. Some patients respond rapidly and completely to treatment with moderate doses of oral prednisone (1 mg/kg/day), others are rather refractory and require much higher doses. If there is no response after 5-7 days, the dose is increased by 50-100% [256,257]. Once the control of the disease is achieved (lack of new lesions, epithelialization of existing erosions and negative Nikolskiy sign [258,259]), the prednisone dosage is decreased in a “logarithmic fashion", i.e. by 10-20% every 7-15 days. Chronic PV patients with disease exacerbation are treated exactly the same way as new patients, i.e. prednisone dose is increased until disease control is achieved and than tapered [256,257].

The first report of administration of glucocorticoids to a pemphigus patient is dated to 1940 [260], i.e. some 25 years earlier than pemphigus antibodies were discovered. Adrenocortical extract was tried for treatment, because it had been noticed that pemphigus is associated with changes in patients’ blood chemistry characteristic of abnormal (deficient) function of the adrenal gland producing cortisone. The synthetic cortisone was introduced to the treatment of pemphigus approximately 10 years later [261]. Prior to the introduction of therapy with oral corticosteroids in the 1950s, the disease had a dismal natural course with a 50% mortality rate at 2 years and 100% mortality rate by 5 years after the onset of the disease. Although there has been a significant decrease in mortality nowadays [262], it remains at a relatively high level of approximately 12% [263], with death being almost invariably related to complications of therapy.

The early adverse effects of systemic glucocorticosteroids that are essentially unavoidable include enhanced appetite, fluid, and salt retention leading to weight gain and neuropsychiatric disorders such as emotional lability, insomnia, irritability, anxiety, depression, euphoria, hyperactivity, and manic episodes. Delayed and insidious adverse effects that depend on cumulative dose include the Cushingoid appearance, hypothalamic-pituitary-adrenal suppression, menstrual disorders, hyperlipidemias, atherosclerosis, cardiovascular events, fatty liver, cataracts, growth retardation, osteoporosis, osteonecrosis, myopathy, muscle cramps and weakness, and skin bruising and thinning [264,265]. Rare and unpredictable adverse effects are glaucoma, pancreatitis and pseu-dotumor cerebri. Treatment of pemphigus patients with corticosteroids can also unmask or aggravate concomitant acne vulgaris, diabetes mellitus hypertension, and peptic ulcer disease.

The goal of pemphigus research is to develop an effective treatment modality that would allow to achieve and maintain clinical remission without the need to use systemic corticosteroids. Although this goal has not been reached yet, a substantial progress was made toward development of steroid-sparing regimens. Based on assumption that glucocorticoids treat pemphigus owing to their immunosuppressive properties, most of the clinical research has been focused on the immunosuppressive therapy. The following is a chronological sequence of the reports of steroid-sparing drugs and therapeutic modalities in the treatment of pemphigus. Immunosuppressive cytotoxic drugs: methotrexate [266], azathioprine [267], cyclophosphamide [268], chlorambucil [269], and mycophenolate mofetil [270]. Immunomodulators: heparin [271], cyclosporine [272], T-cell immunocorrection via photophoresis [273], high-dose intravenous γglobulin, i.e. IVIg [274], rituximab [275] and daclizumab [276]. Anti-inflammatory drugs: gold [277], dapsone [278], doxycycline [279], tetracycline [280], minocycline [281], tranilast [282], and thalidomide [283]. Extracorporeal autoantibody eliminations: plasmapheresis [284], plasma exchange [285], hemocarbofiltration [243], and protein A immunoadsorption [286].

Unfortunately, these therapies do not allow reliable control of acute pemphigus without systemic glucocorticosteroids, indicating that in addition to immunosuppression the therapeutic action of corticosteroids in pemphigus includes other mechanisms, such as direct anti-acantholytic effect on keratinocytes. Moreover, although there is a bulk of evidence that PV is predominantly a Th2-type autoimmune disease, at least with regard to the anti-Dsg 3 antibody production [113,144,159,287], the data on the mechanisms of immunomodulatory action of glucocorticosteroids counterintuitively demonstrate that these drugs foster Th2 polarization of CD4^+^ T cells [288-290].

Direct anti-acantholytic effects of the corticosteroids methylprednisolone and hydrocortisone on keratinocytes were discovered in *in vitro* experiments, in which high doses of these drugs blocked PV IgG-induced acantholysis [291,292]. Because antibody-producing cells were not present in cultures, these drugs could not exhibit their anti-acantholytic effects by way of acting upon lymphocytes. Subsequent *in vivo* experiments demonstrated that administration of methylprednisolone significantly decreased the extent of acantholysis in the epidermis of 3-5-day-old nude mice injected with PV IgG [214]. This was in keeping with the clinical observations that blistering in pemphigus patients stops within 24-48 hrs after initiation of a high dose, “pulse” therapy with methylprednisolone or dexamethasone [293-296], while the major decline in autoantibody titers occurs 3-4 weeks after initiation of glucocorticoid therapy [297]. It is well known that pemphigus therapy improves disease earlier than decreasing the antibody titers [298].

Also, local administration of a 0.05% clobetasol propionate cream can initially control cutaneous lesions in mild cases of PV [299]. The direct effects of corticosteroids that can protect keratinocytes from PV IgG may include alterations in gene expression, as revealed by a DNA microarray assay [214]. PV IgG downregulated and methylprednisolone upregulated expression of the genes encoding the keratinocyte adhesion molecules Dsg 3 and periplakin, regulators of cell cycle progression and apoptosis, differentiation markers, protein kinases and phosphatases, serine proteases and their inhibitors, and some other genes. Furthermore, methylprednisolone blocked phosphorylation of Dsg 3, E-cadherin, and β- and γ-catenins induced by PV IgG [214].

These pharmacologic effects of methylprednisolone help explain the dose-dependent therapeutic action of corticosteroids in pemphigus patients. It is well known that extremely high corticosteroid doses are sometime required to attain control of acantholysis in the acute stage of disease. Thus, in addition to their immunosuppressive and anti-inflammatory actions in pemphigus, glucocorticoids can also regulate adhesion and viability of keratinocytes through a combination of their genomic and non-genomic effects.

Historically, the earliest attempt to treat pemphigus patients by protecting the target cells (keratinocytes) from the PV IgG-induced damage was made using the proteinase inhibitors aprotinin (Contrykal) and ε-aminocaproic acid [242]. A few years later, Dobrev et al. [300] administered *p*-aminomethylbenzoic acid. In both studies, the addition of protease inhibitors allowed to achieve therapeutic effects at lower doses of systemic corticosteroids, thus decreasing the risk for adverse reactions. More recently, TNFa inhibitors have been tried.

Clinical benefit in PV was reported for etanercept (Enbrel) [301-303] and infliximab (Remicade) [304,305], and for adalimumab (Humira) in IgA pemphigus [306]. The p38 MAPK inhibitor KC-706 was used in a multicenter, open-label trial that had to be aborted due to severe adverse reactions. According to Dr Rubenstein's report at the JC Bystryn pemphigus & pemphigoid Meeting [307], KC-706 had been administered orally to 15 patients with PV. One half of the patients experienced a partial response to treatment, while the remaining patients either failed to improve or experienced worsened disease.

Oral nicotinamide (niacinamide) is often used to treat pemphigus patients [280,281,308,309]. Although the exact mechanism of its therapeutic action in pemphigus remains unknown, the efficacy of 4% nicotinamide gel in the treatment of cutaneous erosions of PV patients in a double-blind, placebo-controlled study [310] suggests that it exerts a pharmacologic effect on target cells. In lesional skin, nicotinamide can stimulate keratinocyte adhesion and facilitate epithelialization through its cholinomimetic action [311] that includes both stimulation of ACh release [312] and inhibition of ACh degradation by acetylcholinesterase [313].

The importance of nicotinergic stimulation for the treatment of pemphigus was first suggested by the report of a PV patient whose disease worsened when he stopped smoking and improved shortly after he resumed smoking [314]. The epidemiologic studies confirmed the beneficial effect of smoking on pemphigus. Brenner et al. [315] reported that 25.9% of 126 patients were smokers vs. 48.5% of controls. According to Sullivan et al. [316], only 15.3% of 59 patients were current or former smokers, compared to 47.4% in the general population. These findings have recently been validated in a study involving 199 patients with PV, 11 with PF, and 205 control subjects [317]. Also, it has been reported that smokers with PV achieve partial remission more frequently than non-smokers at the end of the 1st year of treatment, and that the number of patients in remission at the end of the 2nd year of therapy is significantly higher for smokers than for non-smokers [318]. The cigarette smoke contains the nicotinergic agent nicotine that not only upregulates epithelialization *in vitro* [131] but also facilitates healing of skin erosions [319]. In addition to stimulation of epithelialization, nicotine may exhibit its therapeutic effect in pemphigus by affecting the immune system.

Experimental studies demonstrated that activation of nAChRs suppresses B-cell activation [320], abrogates phytohemagglutinin-dependent upregulation of TNFα and IFNγ receptors in T cells [321], inhibits expression of the TNFa, IL-6 and IL-18 genes, and upregulates IL-10 production in macrophages [322]. Furthermore, the nicotinergic signaling facilitates T-cell polarization toward Th1 lineage, inhibits Th17 differentiation, and upregulates Tregs [323,324]. Altogether, these immunopharmacologic effects of nicotine may be able to correct the immune dysregulation characteristic of pemphigus (Figure 5).

The perspective for the development of steroid-sparing therapy employing cholinergic drugs is very promising, because cholinergic agonists of both nicotinic and muscarinic classes have already shown their therapeutic activities in pemphigus patients. The nAChRs in keratinocytes can be directly activated by pyridostigmine bromide (Mestinon) [325]. Besides its underappreciated nicotinergic action, pyridostigmine bromide is reversible acetylcholinesterase inhibitor [326] that can elevate tissue levels of auto/paracrine ACh, thus augmenting signaling through both muscarinic and nicotinic pathways in the cells secreting ACh, like human keratinocytes [327]. Pyridostigmine bromide has been shown to antagonize the effects of PV antibodies in both *in vitro* [328] and *in vivo* experiments [329]. Most importantly, a clinical trial of Mestinon in the treatment of eight pemphigus patients brought encouraging results [330]. Three patients showed a very good response, and five patients did not show any significant improvement. One patient was able to discontinue glucocorticosteroids and immunosuppressive medications, and control the disease using Mestinon only.

In contrast to glucocorticosteroids and protease inhibitors that can only block but not reverse acantholysis [291,331], muscarinic agonists both prevented cell detachment and restored the integrity of keratinocyte monolayers exposed to PV IgGs, the serine proteinase trypsin or the calcium chelator EDTA [130]. These observations indicate that the muscarinic effects stem from activation of the epithelialization program that comprises both the adhesive and the migratory functions of keratinocytes (reviewed in [55,198]). Consistent with the ability of the cholinomimetic carbachol to prevent acantholysis and skin blistering in the neonatal athymic nude mice with passively transferred PV IgGs [329], a double-blind, placebo-controlled study showed therapeutic activity of the muscarinic agonist pilocarpine 4% gel applied to skin erosions of PV patients [332].

Pilocarpine preferentially binds to and activate the M_1_ molecular subtype of keratinocyte mAChRs [333] that has been recently found to be specifically targeted by PV antibodies [49]. Pilocarpine blocks PV IgG-induced phosphorylation of p 120- and β-catenins in keratinocytes, because it elevates both serine/threonine and tyrosine phosphatase activities [215]. Furthermore, the anti-acantholytic activity of pilocarpine synergizes with that of the a7 nAChR agonist AR-R17779 that both activates tyrosine phosphatase and inhibits Src [215]. Taken together, these findings identify novel paradigm of regulation of signaling kinases associated with cholinergic receptors and provide mechanistic explanation of therapeutic activity of cholinomimetics in PV patients.

A large variety of steroid-sparing effects reported thus far in the literature suggests that it should be possible to replace corticosteroids by combining the steroid-sparing drugs and/or treatment modalities that can provide for simultaneous inhibition of antibody production and protection of keratinocytes from autoantibody action. Unfortunately, such putative combination has not been devised yet, though a combination of rituximab and IVIg allows to treat certain PV patients without corticosteroids [334]. Most recently, it has been reported that the mTOR inhibitor sirolimus (Rapamune, Rapamycin®) combined with IVIg allowed rapid and complete withdrawal of systemic glucocorticosteroids in a PV patient who developed disease exacerbation that could not be controlled with 40 mg/day of prednisone [120]. Sirolimus is a naturally occurring lipophilic microcyclic lactone isolated from *Streptomyces hygroscopicus* discovered at Rapa Nui (Easter Island). It binds to immunophilin and FK binding protein-12.

The sirolimus-FKBP-12 complex targets the 290 kD serine-threonine kinase of the phosphoinositide 3-kinases/Akt pathway termed mTOR [335]. Sirolimus exhibits potent immunosuppressive activity due to suppression of T- and B-cell activation and IL-2- and IL-4-dependent proliferation via inhibition of new ribosomal protein synthesis and arrest of the G1-S phase of the cell cycle [336]. In PV, the immunosuppressive action of sirolimus may become therapeutic when it is taken for a period of time. The rapid healing of the skin lesion in the reported case of PV patient in the acute stage of disease [120], however, suggests that it had a direct effect on keratinocytes that protected them from autoantibody action.

As already mentioned, the mTOR pathway is activated in keratinocytes exposed to PV IgG and mTOR inhibition prevents acantholysis in the murine passive transfer model of PV IgG [190]. Additionally, sirolimus may prevent damage of keratinocytes and enforce their adhesive function by inhibiting reorganization of the actin cytoskeleton and phosphorylation of focal adhesion proteins, and upregulating E-cadherin expression [337,338]. The clinical trial of sirolimus in pemphigus patients is currently underway at University of California Irvine (http://clinicaltrials.gov/ct2/show/NCT01313923).

In conclusion, corticosteroids remain an essential component of pemphigus treatment. Early and aggressive use of corticosteroids is required to decrease the duration of treatment and avoid relapses. Adjuvant drugs allow a decrease in the total dose of corticosteroids. The natural course of pemphigus has improved with new therapies. Cholinomimetics can achieve a steroid-sparing effect in pemphigus patients by both stimulating epithelialization and inhibiting autoimmune aggression. The dualistic pharmacologic action of sirolimus that affects both effectors of autoimmunity and target cells apparently mediates its therapeutic effect in pemphigus. Further elucidation of the molecular mechanisms mediating aberrant signaling along the mTOR pathway in PV should improve our understanding of the pathogenesis and lead to novel therapeutic approaches for the development of steroid-free treatment of pemphigus.

### Summary

1. Recent advances of knowledge on pemphigus autoimmunity scrutinize old dogmas, resolve controversies, and open novel perspectives for treatment (Table II).
2. The initial insult is sustained by the autoanti- bodies to the cell-membrane receptor antigens triggering the intracellular signaling by Src, EGFRK, PKC, phospholipase C, mTOR, p38 MAPK, other tyrosine kinases, and calmodulin that cause basal cell shrinkage and ripping desmosomes off the CM.
3. Autoantibodies synergize with the effectors of apoptotic and oncotic pathways, serine proteases and inflammatory cytokines to overcome the natural resistance and activate the cell death program in keratinocytes.
4. The process of keratinocyte shrinkage/detachment and death via apoptosis/oncosis has been termed apoptolysis to emphasize that it is triggered by the same signal effectors and mediated by the same cell death enzymes.
5. Although a high-dose, long-term systemic glucocorticoid therapy remains the mainstay of current treatment of patients with PV or PF, causing severe adverse effects, a substantial progress has been made toward development of steroid- sparing therapies combining the immunosuppressive and direct anti-acantholytic effects.
6. The onset of acantholysis in drug-induced pemphigus in patients taking angiotensin- converting enzyme inhibitors, such as captopril, apparently involves a drop in the concentration of auto/paracrine ACh that sustains keratinocyte shape and cohesiveness, due to a strong upregulation of the ACh degrading enzyme acetylchol- inesterase [351].

**Table II:** Hypotheses and realities in the knowledge of pemphigus.

Hypotheses	Realities
The epidermal integrity is mediated exclusively by the Dsg 1 and 3 adhesion molecules	Neither Dsg 1 nor Dsg 3 can solely sustain keratinocyte adhesion in epidermis. Patients with striate palmoplantar keratoderma featuring N-terminal deletion in Dsg 1 do not develop skin blisters [350], In turn, the conditional Dsc3^nu^” mutant mouse develops PV phenotype despite the presence of intact Dsg 3 [43]. If the integrity of epidermis would rely exclusively on Dsg 1 and 3, the epidermis should disintegrate to a single cell suspension in the PV patients who develop both anti-Dsg 1 and 3 antibodies (Figure 2)
Acantholysis is PV is caused by steric hindrance of Dsg 3 by autoantibodies	Electron microscopic studies of limited acantholysis produced by anti-Dsg 3 antibody in murine epidermis revealed that steric hindrance of Dsg 3 leads to a desmosomal split without keratin retraction [84,85], The ultrastructural changes in the skin of PV patients are quite different. Desmosomes remain intact till the late stages of acantholysis when they are cleaved behind the desmosomal plaque, due to shearing forces produced by collapsing cells, and float free in the intercellular space [55,86-90], Acantholysis is PV results from PV antibody-dependent signaling events collectively described by the term apoptolysis [1]
Clinical and histological features of PF and PV can be reproduced solely by Dsg 1 and 3 antibodies, respectively	The experiments using the Dsgl-Ig and Dsg3-Ig chimeras that absorbed out all disease causing pemphigus antibodies, thus giving a rise to a notion that anti-Dsg 1/3 antibodies are the sole cause of pemphigus, were flawed by the presence of non-Dsg antibodies (Figure 3)
An interplay between Dsg 1 and 3 antibodies determines the mucocutaneous phenotype in patients with autoimmune pemphigus	PF patients can develop antibodies against both Dsg 1 and Dsg 3 [108,116,117], and the Dsg 1/3 antibody pattern does not match the predicted morphologic phenotype ofPV [96,118,119]
The titers of anti-Dsg 1 or 3 antibodies correlate closely with the severely of the disease	The Dsg 1/3 antibody titers do not correlate with disease activity [91-93], While Dsg 3 antibody can be absent in PV patients in active stage of disease, it can be present in PV patients during remission [94-96] as well as in healthy subjects and patients with irrelevant medical conditions [97-109]
The sera of patients with autoimmune pemphigus contain autoantibodies only to the Dsg 1/3 targets	More than 50 organ-specific and non-organ-specific proteins have been reported to date as specific targets for autoantibodies produced by PV and/or PF patients (Table I)
Systemic corticosteroids treat pemphigus patients exclusively by inhibiting autoantibody production	The therapeutic effect of “pulse” therapy with methylprednisolone commences within a few days, whereas autoantibody titers decline within 3-4 weeks [294-296], The rapid therapeutic effect is apparently mediated by direct anti-acantholytic action of glucocorticosteroids that protects keratinocytes from an autoantibody-induced damage [214]
Paraneoplastic pemphigus (PNP) is a variant of classical pemphigus	PNP is not related to PV and PF, but represents a clinical variant of the paraneoplastic autoimmune multiorgan syndrome (PAMS) in which patients, in addition to small airway occlusion, may display a spectrum of at least five clinical variants, i.e. pemphigus like (a.k.a. PNP), pemphigoid like, erythema multiforme like, graft vs. host disease like, and lichen planus like [10,12,13]
